# Effect of MW-assisted roasting on nutritional and chemical properties of hazelnuts

**DOI:** 10.3402/fnr.v59.28916

**Published:** 2015-12-17

**Authors:** Fatih Kalkan, Sai Kranthi Vanga, Yvan Gariepy, Vijaya Raghavan

**Affiliations:** Department of Bioresource Engineering, Faculty of Agriculture and Environmental Studies, McGill University, Sainte-Anne-de-Bellevue, QC, Canada

**Keywords:** microwave roasting, microwave-assisted hot air roasting, antioxidant activity, resistant starch, protein concentration

## Abstract

In order to enhance the flavor, texture, color, and appearance of hazelnuts, they are roasted during postharvest processing. In this study, raw hazelnuts (*Corylus avellan*a L.) were roasted using microwave (MW) and MW-assisted hot air methods under various roasting conditions. The hazelnuts roasted were then examined to determine the percent DPPH radical scavenging activity, antioxidant capacity, total phenolic content, resistant starch, non-resistant starch, total starch, and protein concentration. The roasting experiments were done using a completely randomized factorial arrangement of two roasting types by three roasting times (9, 15, and 21 min) by three roasting temperatures (70, 90, and 110°C) using three replications within each experiment. These roasting methods were found to yield significant differences in antioxidant capacity, total phenolic content, resistant starch, non-resistant starch, and protein concentration between MW and MW-assisted hot air roasting processes, while no difference was found in percent DPPH radical scavenging activity and total starch. The results obtained may be of great importance to the food research community and industrial hazelnut roasting technologies.

Most of the world's hazelnuts (*Corylus avellana* L.) are grown in Turkey (72%; 660,000 tonnes), and almost all are produced in the Black Sea region alone on more than 400,000 ha. Turkey is followed by Italy, the United States, and Spain in hazelnut production (1). The hazelnut is a tree nut; other tree nuts include the almond, Brazil nut, cashew, hazelnut, macadamia nut, pecan, pine nut, pistachio, and walnut. In the world market, tree nuts are segmented into two main groups, in-shell and shell tree nuts. In-shell nuts are the ones that are produced and marketed with the shell intact. However, tree nuts used in the food industry are mostly shelled, where the external shell is removed from the nut using any mechanical processing methods. Shelled nuts are then exposed to various processing methods including blanching, dicing, coating, roasting, or grinding (and combinations of the above methods) to get a properly prepared product and increase consumer appeal (2).

Hazelnuts are roasted for several reasons, primarily to enhance the flavor, texture, color, and appearance of the product significantly. In a few cases various treatments may also ensure the destruction of any toxins or allergens that may exist in the hazelnut. (2–4). Changes in flavor, texture, and color during the roasting process are primarily related to drying and non-enzymatic browning of the hazelnuts (5–7). Non-enzymatic browning is caused by heat treatments and generally includes several kinds of reactions including the Maillard reaction, caramelization, chemical oxidation of phenols, and maderization (8). Kim et al. reported that brown color, antioxidant activity, and flavor in agricultural products increases due to the Maillard reaction during the roasting process (9). Kim et al. also reported that roasting small black soybeans at higher temperatures and longer roasting times caused an increase in the degree of browning and antioxidant activity (9). The use of natural antioxidants has increased recently due to concerns regarding the safety of synthetic ones, even though several synthetically obtained antioxidants such as BHT (butylated hydroxytoluene), BHA (butylated hydroxyanisole), propyl gallate (PG), and ethyl protocatechuate (EP) are extensively used in the food industry and the human diet (10). Overall, the nutritional as well as other properties of agricultural products may be improved by application of a thermal treatment such as drying or roasting (2–4, 9, 11–14).

Scientific reports on the physicochemical and nutritional properties of hazelnuts roasted by microwave (MW) and MW-assisted hot air are very scarce, and the current study will help to fill this gap in the literature. Hence, MW and MW-assisted hot air roasting techniques were applied to raw hazelnuts, which were then examined to determine the percent DPPH radical scavenging activity, antioxidant capacity, total phenolic content, resistant starch, non-resistant starch, total starch, and protein concentration.

## Materials and methods

### Reagents

Materials included methanol (Fisher Chemicals, A452-4, UK), ethanol (Fisher Chemicals, A962-4, UK), 2,2-diphenyl-1-picrylhydrazyl (DPPH) (Sigma-Aldrich, D9132, USA), BHA (Sigma-Aldrich, B1253, USA), gallic acid (Sigma-Aldrich, G7384, USA), Folin–Ciocalteu's phenol reagent (Sigma-Aldrich, F9252, USA), anhydrous sodium carbonate (Fisher Chemicals, S263-1, UK), maleic acid (Sigma-Aldrich, M0375, USA), sodium hydroxide (American Chemicals, S290, USA), calcium chloride dihydrate (Fisher Chemicals, C79-500, UK), sodium azide (Sigma-Aldrich, S2002, USA), potassium hydroxide (Fisher Chemicals, S71978, UK), glacial acetic acid (Fisher Chemicals, A38-500, UK), Megazyme resistant starch assay kit (Megazyme International Ireland Ltd, Bray, Ireland), and Pierce™ BCA protein assay kit (Pierce, Rockford, IL, USA).

### Sample preparation

Hazelnuts (*C. avellana* L., Jumbo Barcelona variety) used in this study were purchased from a local market (Hazelnut Growers of Oregon, USA) as in-shell nuts. They were cracked by hand, and broken kernels were then removed from the bulk. Before the experiment, the initial moisture content of the hazelnuts was determined (15); it was found to be 5.7% d.b.

### Automated MW-assisted thermal system

The roasting process was performed using an automated MW-assisted thermal system designed in the post-harvest technology laboratory (Macdonald Campus, McGill University). The automated MW-assisted thermal system was capable of both MW and MW-assisted hot air roasting processes. This system consisted of an air blower, air heaters, an MW chamber with balance, magnetron device, MW meter, waveguides, temperature-measuring devices such as thermocouples and fiber optic cables, and a data acquisition system. The MW generator operated at 2,450 MHz with variable power from 0 to 750 W. The thermocouples, fiber optic cables (Nortech EMI-TS series, Quebec City, Canada), and balance were connected to an Agilent 34970A data acquisition system (Agilent Technologies, Santa Clara, CA, USA), and this system was also connected to a computer.

### Experimental procedure

#### MW experiments

MW experiments were carried out using a completely randomized factorial arrangement of two roasting types by three roasting times by three roasting temperatures using three replications within each experiment. Hazelnut samples of 90±1 g were subjected to MW and MW-assisted hot air roasting processes for 9, 15, and 21 min at temperatures of 70, 90, and 110°C. MW power density was maintained at 1.1 W/g during all experiments. Samples were placed in a single layer on the mesh sample holder, which was attached to a balance. In the MW-assisted hot air roasting process, the air was heated by three 2-kW heaters and continuously circulated through the mesh by the blower, which had 0.2 kW of power and was placed below the roasting cavity. The temperature of the hazelnuts was measured during the roasting process with the help of an optical fiber probe.

#### Hazelnut extraction

Two grams of hazelnut powder was extracted in 25 ml of 99.9% (v/v) methanol at room temperature by stirring for 24 h at 175 rpm and then filtering through Whatman Grade 4 filter paper Whatman (Clifton, NJ, USA). These extracts were analyzed for their antioxidant activity, total phenol content, and protein concentration.

#### Percent DPPH radical scavenging activity and antioxidant capacity

Percent DPPH radical scavenging activity and antioxidant capacity were determined using DPPH free radical, which was a modification of the method from Ohinishi et al. (16). An aliquot of hazelnut powder extract solution (270 µl) was added to 1,620 µl of DPPH solution (0.00394 g DPPH per 100 ml of 99.9% (v/v) methanol). After 20 min of incubation in the dark, the absorbance values were recorded at 517 nm (Ultrospec 2100 pro, Biochrom Ltd., Cambridge, UK). The reference was 99.9% (v/v) methanol. Free radical scavenging activity on DPPH radicals was expressed as percent (%) inhibition:

1 $$\mathrm{Scavenging}\mathrm{activity}(\%)=100\times \left(\frac{\mathrm{Ab}s_{\mathrm{blank}}^{517\mathrm{nm}}-\mathrm{Ab}s_{\mathrm{sample}}^{517\mathrm{nm}}}{\mathrm{Ab}s_{\mathrm{blank}}^{517\mathrm{nm}}}\right)$$

So as to express antioxidant capacity, a standard curve of BHA was obtained from DPPH radical scavenging activity (%) plotted against various BHA concentrations. The concentrations of BHA solution were 2.5, 5, 10, 15, 20, 25, and 30 µg/ml of 99.9% (v/v) methanol. All data were the average of triplicate analyses.

#### Determination of total phenolic content

Total phenolic content was determined according to the Folin–Ciocalteu procedure (17) with minor modifications. The hazelnut powder extract solution (320 µl) and standard solution of gallic acid (320 µl) (50, 100, 150, 200, 250, 300, and 350 µg/ml of 99.9% (v/v) methanol) were added to a 15 ml volumetric capped tube. A reagent reference was prepared by using 99.9% (v/v) methanol, 10% (v/v) Folin–Ciocalteu reagent, 7.5% (v/v) Na_2_CO_3_ solution, and deionized water. To the samples and standard solutions were added 1,280 µl of 10% (v/v) Folin–Ciocalteu reagent and 800 µl of 7.5% (v/v) Na_2_CO_3_ solution in succession; the mixtures were then shaken for 10 sec. After 3 min, 1,600 µl of deionized water was added to the mixtures and they were shaken for 10 sec. The mixtures were incubated for 30 min in a 40°C water bath and then cooled for 15 min at ambient temperature in the dark. The absorbance against the reagent reference was determined at 765 nm. The total phenolic content was expressed as gallic acid equivalents (GAE) in milligrams per gram of dry sample, using a standard curve generated with gallic acid. All data were the average of triplicate analyses.

#### Determination of resistant starch, non-resistant starch, and total starch

Resistant starch, non-resistant starch, and total starch contents were analyzed using the Megazyme resistant starch assay kit.

In brief, 100±5 mg of sample was weighed individually into screw-cap tubes. Pancreatic α-amylase solution (10 mg mL^−1^) containing 4 mL of amyloglucosidase (AMG) (3 U mL^−1^) was then added to the tubes, which were subsequently incubated at 37°C while being continuously shaken (200 strokes/min) for exactly 16 hr. After incubation, 4 mL of ethanol (99.9% v/v) was added to the tubes to aid dispersion, with vigorous stirring on a vortex mixer; the tubes were then centrifuged at 1,500 g for 10 min. The supernatant was decanted and kept for nonresistant analysis. The pellets were resuspended in 2 mL of ethanol (50% v/v) with vigorous stirring on a vortex mixer; another 6 mL of ethanol (50% v/v) was added, and the mixture was centrifuged again at 1,500 g for 10 min. This suspension and centrifugation process was repeated once more.

##### Determination of resistant starch

The pellets were resuspended with 2 mL of 2 M KOH and stirred for 20 min in an ice bath with a magnetic stirrer. Next, 8 mL of 1.2 M sodium acetate buffer (pH 3.8) was added to the tubes as the mixture was stirred by the magnetic stirrer and then 0.1 mL of AMG (3 U mL^−1^) was immediately added. The mixture was stirred well and kept in a water bath at 50°C for 30 min. After incubation, the tubes were directly centrifuged at 1,500 g for 10 min. A 0.1 mL aliquot of the supernatant was treated with 3.0 mL glucose oxidase/peroxidase (GOPOD) reagent and incubated at 50°C for 20 minutes. Sodium acetate buffer (0.1 M, pH 4.5) and glucose (1 mg/mL in 0.2% benzoic acid) were used as a blank and glucose standard, respectively. The absorbance was measured using a spectrophotometer at 510 nm. Resistant starch (g/100 g sample) was calculated using an equation from the kit manual. The analyses were carried out twice.

##### Determination of non-resistant starch

The supernatant obtained from centrifugation was adjusted to 100 mL with 0.1 M sodium acetate buffer (pH 4.5). A 0.1 mL aliquot of supernatant was incubated with 10 µL of dilute AMG solution (3 U mL^−1^) in 0.1 M sodium maleate buffer (pH 6.0) for 20 min at 50°C and followed by a second incubation with 3.0 mL of GOPOD reagent for a further 20 min at 50°C. The absorbance was recorded at 510 nm against a reagent blank. Non-resistant starch (g/100 g sample) was calculated using an equation from the kit manual. The analyses were carried out twice.

Total starch was obtained from the sum of resistant starch and non-resistant starch, as seen from Equation 8.

2 Total starch=resistant starch+non-resistant starch

#### Determination of protein concentration

Protein concentration was analyzed using a Pierce BCA protein assay kit standardized with bovine serum albumin (BSA).

## Results and discussion

The percent DPPH radical scavenging activity, antioxidant capacity, total phenolic content, resistant starch, non-resistant starch, total starch, and protein concentration were determined for both unroasted and roasted hazelnuts (see Table 1). After determining these properties, we examined their differences before and after the roasting process.

**Table 1:** Properties of unroasted hazelnuts

Properties	Values
Percent DPPH radical scavenging activity	50.88±4.23*
Antioxidant capacity, mg of BHA/g of dry sample	0.48±0.04
Total phenolic content, mg of GAE/g of dry sample	0.74±0.08
Resistant starch, % d.b.	1.52±0.01
Non-resistant starch, % d.b.	1.19±0.07
Total starch, % d.b.	2.71±0.08
Protein concentration, µg of BSA/ml of extract	154.99±3.32

* Mean±standard deviation. BHA, butylated hydroxyanisole; BSA, bovine serum albumin; GAE, gallic acid equivalents; DPPH, 2,2-diphenyl-1-picrylhydrazyl.

### Effect of MW and MW-assisted hot air roasting process on percent DPPH radical scavenging activity and antioxidant capacity

In order to evaluate the ability of antioxidants to the scavenge free radicals, the percent DPPH radical scavenging assay is extensively used. Absorbance values recorded at 517 nm decrease with progression of the reaction between antioxidant molecules and DPPH radicals. A lower absorbance value means higher antioxidant activity of an extract in terms of its hydrogen atom-donating capacity, indicating that it is, therefore, more potent (18, 19). Table 2 presents the percent DPPH radical scavenging activity of hazelnuts roasted by MW and MW-assisted hot air roasters. Changes in the concentrations of DPPH radicals due to the scavenging ability of the roasting temperatures of 70, 90, and 110°C were not statistically substantial for 9 min of the roasting time in MW roasting process, whereas it was statistically significant in the MW-assisted hot air roasting process (*p*<0.01). On the other hand, in both MW and MW-assisted hot air roasting processes, there was a statistically significant difference among percent DPPH scavenging activities at the roasting temperatures for both the 15- and 21-min roasting times (*p*<0.01). Percent DDPH radical scavenging activity, which was 50.88 for unroasted hazelnuts, linearly increased with the roasting temperature up to 110°C for each roasting time in the MW-assisted hot air roasting process, whereas no similar trend was observed in the MW roasting process. In the MW roasting process, percent DDPH radical scavenging activity first increased between unroasted hazelnuts and hazelnuts roasted at 70°C for 9 min and then decreased with an increase in the roasting temperature. The highest percent DDPH radical scavenging activity was obtained at a roasting temperature of 90°C for the 15- and 21-min roasting times in the MW roasting process. As seen in Table 2, when hot air was applied in combination with MW to the hazelnuts, the percent DDPH radical scavenging activity increased up to the final roasting temperature (110°C) at all the roasting times. It may be concluded that while higher temperatures such as 110°C may have has a negative effect on percent DPPH scavenging activity in the MW roasting process, they had a positive effect when the MW-assisted hot air roasting process was used. Overall, the highest percent DPPH scavenging activity was obtained at a roasting temperature of 90°C with a 21-min roasting time, whereas the lowest one was obtained at 110°C with a 9-min roasting time in the MW roasting process. In the MW-assisted hot air roasting process, a 110°C roasting temperature presented the strongest percent DPPH scavenging activity when processed for 15 min; the 70°C roasting temperature presented the weakest percent DPPH scavenging activity at a roasting time of 9 min. Many previous studies have shown that thermal processing such as drying and roasting preserves health benefits by enhancing antioxidant activity, which supports the results of this study (9, 12–14). It may be said that it is possible to improve the nutritional quality of hazelnuts with percent DPPH scavenging activity by MW and MW-assisted hot air roasting processes. In one of the studies mentioned above, Kim et al. compared the antioxidant effects of small black soybeans under different roasting process conditions using DPPH and ABTS assays (9). They reported that roasted small black soybeans exhibited significantly higher antioxidant activity than unroasted small black soybeans in the DPPH and ABST assays.

**Table 2:** Percent DPPH scavenging activities of hazelnuts in MW and MW-assisted hot air roasting processes

		Temperature, °C			
			
Roasting type	Time, min	70	90	110	Mean
MW	9	67.78±8.75dδ	64.74±6.12d	63.24±9.82d	65.25a*
	15	86.82±5.37d	87.79±6.93e	75.59±7.19f	83.37b
	21	82.55±5.27d	95.10±7.70e	88.69±5.42f	88.78c
	Mean	79.05	82.51	75.84	79.14A£
MW-assisted hot air	9	62.98±8.05hδ	70.76±10.34i	78.66±8.47j	70.80d*
	15	69.85±5.14h	89.82±1.92i	94.78±0.77j	84.82e
	21	72.82±2.84h	87.54±4.32i	93.53±1.91j	84.63e
	Mean	68.55	82.71	88.99	80.08A

Mean±standard deviation. MW, microwave.

* Means in the same column followed by the same lowercase letter are not significantly different (*p*<0.01) by Duncan's multiple range test.

δ Means in the same row followed by the same lowercase letter are not significantly different (*p*<0.01) by Duncan's multiple range test.

£ Means in the same column followed by the same uppercase letter are not significantly different (*p*<0.01) by Duncan's multiple range test.

The changes in antioxidant capacity and the corresponding values of roasted hazelnuts are presented in Table 3. While unroasted hazelnuts have 0.48 mg of BHA/g dry sample of antioxidant capacity, the roasting process resulted in increased antioxidant capacity in both MW and MW-assisted hot air processes under the roasting conditions studied. The highest antioxidant capacity was obtained at 90°C for all the roasting times in the MW roasting process (*p*<0.01). By contrast, a linear increase in antioxidant capacity in the MW-assisted hot air process was observed with increased roasting temperature from 70 to 110°C at all the roasting times (*p*<0.01). The antioxidant capacities of hazelnuts that were roasted in an MW roaster were mostly higher than those of hazelnuts that were roasted in an MW-assisted hot air roaster under all the roasting conditions studied (*p*<0.01). It may be inferred that the rise in antioxidant capacity may be negatively affected by hot air applied with MW in the hazelnut roasting process. The roasting temperature of 90°C yielded the highest antioxidant capacity (1.25 mg of BHA/g dry sample) at a 21-min roasting time using the MW roasting process, while the lowest antioxidant capacity (0.60 mg of BHA/g dry sample) was obtained by roasting at 70°C for 9 min. Whereas the highest antioxidant capacity (1.08 mg of BHA/g dry sample) was obtained at 110°C for 15 min using the MW-assisted hot air roasting process, the roasting temperature of 70°C presented the lowest antioxidant capacity (0.40 mg of BHA/g dry sample) at 9 min of the roasting time. It is noteworthy that the lower temperature with lower roasting time may cause a decrease in antioxidant capacity using the MW-assisted hot air roasting process. Likewise, the antioxidant capacity of 0.40 mg of BHA/g dry sample, obtained using the MW-assisted hot air roasting process at 70°C for 9 min, was lower than antioxidant capacity of unroasted hazelnuts. We have not found any information concerning the effects of MW hazelnut roasting conditions on antioxidant capacity. Nevertheless, in prior research, the antioxidant capacity of cocoa kernels subjected to different roasting treatments was determined by Summa et al. (20). Their research showed that the roasting time caused an increase in the antioxidant capacity of kernels, which supported our results.

**Table 3:** Antioxidant capacities of hazelnuts in MW and MW-assisted hot air roasting processes (mg of BHA/g of dry sample)

		Temperature, °C			
			
Roasting type	Time, min	70	90	110	Mean
MW	9	0.60±0.08dδ	0.93±0.10e	0.91±0.15e	0.82a*
	15	1.02±0.17d	1.20±0.05e	0.92±0.22d	1.04b
	21	0.95±0.12d	1.25±0.11e	1.01±0.06d	1.07c
	Mean	0.85	1.13	0.95	0.98A£
MW-assisted hot air	9	0.40±0.07dδ	0.60±0.09e	0.66±0.07e	0.55a*
	15	0.55±0.08d	0.74±0.02e	1.08±0.01f	0.79b
	21	0.76±0.03d	0.92±0.05e	0.99±0.02f	0.89c
	Mean	0.57	0.75	0.91	0.75B

Mean±standard deviation. BHA, butylated hydroxyanisole; MW, microwave.

* Means in the same column followed by the same lowercase letter are not significantly different (*p*<0.01) by Duncan's multiple range test.

δ Means in the same row followed by the same lowercase letter are not significantly different (*p*<0.01) by Duncan's multiple range test.

£ Means in the same column followed by the same uppercase letter are not significantly different (*p*<0.01) by Duncan's multiple range test.

### Effect of MW and MW-assisted hot air roasting process on total phenolic content

Table 4 presents the total phenolic content of roasted hazelnuts, which are in accordance with published data (21, 22). Although no statistical differences were presented by the roasting temperature on the total phenolic content at the 15-min roasting time using the MW roasting process, the roasting temperature statistically affected the total phenolic content at the 9- and 21-min roasting times (*p*<0.01). The total phenolic content was also statistically affected by the roasting temperature using the MW-assisted hot air roasting process at all the roasting times (*p*<0.01). While the maximum total phenolic content was presented as 1.37 mg of GAE/g dry sample at 90°C with a 21-min roasting time using the MW roasting process under the various roasting conditions studied, it was obtained as 1.53 mg of GAE/g dry sample at 110°C with a 9-min roasting time using the MW-assisted hot air roasting process. Hot air applied with an MW had a more positive effect on the total phenolic content at the 9-min roasting time for all roasting temperatures, and this effect then decreased with an increase in the roasting time using the MW-assisted hot air roasting process. The total phenolic content of hazelnuts roasted in the MW roaster were mostly lower than for hazelnuts roasted in the MW-assisted hot air roaster under all roasting conditions (*p*<0.01). Potent statistical correlations were also observed between the total phenolic content and percent DPPH scavenging activity (*p*<0.01, *p*<0.01) and antioxidant capacity (*p*<0.01, *p*<0.05) in the MW and MW-assisted hot air roasting processes, respectively.

**Table 4:** Total phenolic contents of hazelnuts in MW and MW-assisted hot air roasting processes (mg of GAE/g of dry sample)

		Temperature, °C			
			
Roasting type	Time, min	70	90	110	Mean
MW	9	0.98±0.17dδ	0.74±0.08e	0.82±0.07e	0.85a*
	15	0.99±0.10d	1.06±0.14d	0.97±0.16d	1.00b
	21	1.00±0.10d	1.37±0.12e	0.99±0.13d	1.12c
	Mean	0.99	1.06	0.93	0.99A£
MW-assisted hot air	9	0.91±0.09dδ	1.39±0.16e	1.53±0.14f	1.28a*
	15	0.79±0.14d	0.92±0.05e	1.35±0.08f	1.02b
	21	0.81±0.04d	0.95±0.08e	1.22±0.13f	1.00c
	Mean	0.84	1.09	1.37	1.10B

Mean±standard deviation. GAE, gallic acid equivalents; MW, microwave.

* Means in the same column followed by the same lowercase letter are not significantly different (*p*<0.01) by Duncan's multiple range test.

δ Means in the same row followed by the same lowercase letter are not significantly different (*p*<0.01) by Duncan's multiple range test.

£ Means in the same column followed by the same uppercase letter are not significantly different (*p*<0.01) by Duncan's multiple range test.

### Effect of MW and MW-assisted hot air roasting process on resistant starch, non-resistant starch, and total starch

The resistant starch, non-resistant starch, and total starch of roasted hazelnuts are given in Tables 5–7). No substantial differences were statistically observed in the levels of resistant starch of roasted hazelnuts over all roasting temperatures and times using both MW and MW-assisted hot air roasting processes. The levels of resistant starch for hazelnuts roasted using the MW roasting process ranged from between 1.22 and 1.48% d.b., which were lower than for unroasted hazelnuts. Among the roasting conditions, the highest level of resistant starch was found at 110°C with a 15-min roasting time, and the lowest level was obtained by roasting at 70°C for 21 min using the MW roasting process. By comparison, the levels of resistant starch for hazelnuts roasted in the MW-assisted hot air roaster varied between 1.34 and 1.54% d.b. The roasting temperature of 110°C yielded the maximum level of resistant starch with the 15-min roasting time, whereas roasting at 90°C for 21 min yielded the lowest level of resistant starch using the MW-assisted hot air roasting process among all roasting conditions. Overall, as seen from Table 5, the levels of resistant starch of hazelnuts roasted using the MW-assisted hot air roaster were higher than those of hazelnuts roasted using the MW roaster under all roasting conditions (*p*<0.01). This trend may be contributed to by applying hot air with an MW. We concluded that the MW roasting process decreased the level of resistant starch compared to unroasted hazelnuts at all the roasting conditions, whereas using the MW-assisted hot air roasting process increased the level of resistant starch compared to that of unroasted hazelnuts at some roasting conditions and caused a smaller reduction than the MW roasting process. In this context, as reported by Pereira and Leonel (23), food processing techniques including sterilizing, drying in ovens, or drying at high temperatures increases the level of resistant starch (24). A study supporting our results also reported that the level of resistant starch of chickpeas and beans decreased with application of a thermal treatment by MW (25). Conversely, unlike the levels of resistant starch, the levels of non-resistant starch were statistically affected by the roasting temperature under some roasting conditions (Table 6). A similar trend was also observed for the level of total starch, since the level of non-resistant starch affected the level of total starch (Table 7). At the higher roasting temperatures and longer times in both MW and MW-assisted hot air roasting process, non-resistant starch particles were easily degraded into sugar, as seen from Table 6.

**Table 5:** Levels of resistant starch of hazelnuts in MW and MW-assisted hot air roasting processes (% d.b.)

		Temperature, °C			
			
Roasting type	Time, min	70	90	110	Mean
MW	9	1.26±0.10dδ	1.33±0.11d	1.39±0.22d	1.33a*
	15	1.38±0.16d	1.30±0.15d	1.48±0.13d	1.39a
	21	1.22±0.36d	1.25±0.18d	1.32±0.10d	1.27a
	Mean	1.29	1.29	1.40	1.33A£
MW-assisted hot air	9	1.40±0.16dδ	1.44±0.14d	1.53±0.22d	1.46a*
	15	1.40±0.20d	1.45±0.13d	1.54±0.16d	1.46a
	21	1.38±0.07d	1.34±0.03d	1.49±0.23d	1.40a
	Mean	1.39	1.41	1.52	1.44B

Mean±standard deviation. MW, microwave.

* Means in the same column followed by the same lowercase letter are not significantly different (*p*<0.01) by Duncan's multiple range test.

δ Means in the same row followed by the same lowercase letter are not significantly different (*p*<0.01) by Duncan's multiple range test.

£ Means in the same column followed by the same uppercase letter are not significantly different (*p*<0.01) by Duncan's multiple range test.

**Table 6:** Levels of non-resistant starch of hazelnuts in MW and MW-assisted hot air roasting processes (% d.b.)

		Temperature, °C			
			
Roasting type	Time, min	70	90	110	Mean
MW	9	1.13±0.40dδ	0.78±0.05d	0.84±0.14d	0.91ab*
	15	1.18±0.06d	0.94±0.16e	0.87±0.14e	1.00a
	21	0.74±0.06d	0.86±0.25d	0.86±0.15d	0.82b
	Mean	1.01	0.86	0.86	0.91A£
MW-assisted hot air	9	0.75±0.05dδ	1.06±0.24e	0.93±0.14de	0.91a*
	15	1.25±0.17d	0.64±0.00e	0.59±0.07e	0.83a
	21	0.76±0.05d	0.57±0.04e	0.61±0.21de	0.65b
	Mean	0.92	0.76	0.71	0.80B

Mean±Standard deviation. MW, microwave.

* Means in the same column followed by the same lowercase letter are not significantly different (*p*<0.01) by Duncan's multiple range test.

δ Means in the same row followed by the same lowercase letter are not significantly different (*p*<0.01) by Duncan's multiple range test.

£ Means in the same column followed by the same uppercase letter are not significantly different (*p*<0.01) by Duncan's multiple range test.

**Table 7:** Levels of total starch in hazelnuts roasted using MW and MW-assisted hot air roasting processes (% d.b.)

		Temperature, °C			
			
Roasting type	Time, min	70	90	110	Mean
MW	9	2.39±0.40dδ	2.11±0.11d	2.23±0.31d	2.24ab*
	15	2.56±0.07d	2.24±0.17e	2.36±0.16e	2.38a
	21	1.96±0.37d	2.11±0.32d	2.18±0.21d	2.08b
	Mean	2.30	2.15	2.25	2.24A£
MW-assisted hot air	9	2.14±0.20dδ	2.50±0.28e	2.47±0.28e	2.37a*
	15	2.65±0.30d	2.09±0.13e	2.13±0.14e	2.29a
	21	2.14±0.10d	1.92±0.07d	2.09±0.27d	2.05b
	Mean	2.31	2.17	2.23	2.24A

Mean±standard deviation. MW, microwave.

* Means in the same column followed by the same lowercase letter are not significantly different (*p*<0.01) by Duncan's multiple range test.

δ Means in the same row followed by the same lowercase letter are not significantly different (*p*<0.01) by Duncan's multiple range test.

£ Means in the same column followed by the same uppercase letter are not significantly different (*p*<0.01) by Duncan's multiple range test.

### Effect of MW and MW-assisted hot air roasting processes on protein concentration

The initial level of protein concentration of unroasted hazelnuts was 154.99 µg of BSA/ml of extract. Table 8 presents the levels of protein concentrations for roasted hazelnuts, which were higher than those of unroasted hazelnuts for all the roasting conditions studied. There were no statistically significant differences among the levels of protein concentrations at the roasting temperatures using the MW roasting process for all the roasting times, however the roasting temperature statistically affected the levels of protein concentrations using MW-assisted hot air roasting process at all the roasting times studied. Such a trend may be contributed to by application of hot air with an MW. As seen from Table 8, while a linear increase was observed in the levels of protein concentrations with increasing roasting temperature using the MW-assisted hot air roasting process at all the roasting times, it was not observed for the MW roasting process except under one condition, which already had no statistically significant effect. The highest protein concentration (243.34 µg of BSA/ml of extract) was obtained using the MW roasting process at 110°C for 21 min; this concentration was 57% higher than the protein concentration of unroasted hazelnuts. The MW-assisted hot air roasting process also increased the protein concentration by 105% at a 110°C roasting temperature and a 21-min roasting time. This increase was for the highest level of protein concentration using the MW-assisted hot air roasting process. In this context, Locatelli et al. reported that the protein content of medium roasted hazelnuts was lower than that of dark roasted hazelnuts, which supported our results (10). Higher levels of protein in roasted hazelnuts compared to unroasted hazelnuts may be explained by the protein digestibility. In their study, Kong and Singh (26) emphasized that the roasting process caused an increase in protein digestibility. In recent studies conducted by Vanga et al. similar results were observed where processing of peanuts increased the protein digestibility (27). Holter and Reid also reported that there was a relationship between level of protein concentration and level of protein digestibility of some forages (28). Overall, levels of protein concentration in hazelnuts roasted by the MW-assisted hot air roaster were higher than the levels of hazelnuts roasted by MW roaster, as seen from Table 8 (*p*<0.01). This trend may be explained by application of hot air with an MW.

**Table 8:** Levels of protein concentration in hazelnuts roasted using MW and MW-assisted hot air roasting processes (µg of BSA/ml of extract)

		Temperature, °C			
			
Roasting type	Time, min	70	90	110	Mean
MW	9	199.47±31.17dδ	187.21±4.89d	185.96±28.61d	190.88a*
	15	214.37±11.10d	219.91±27.70d	205.07±4.12d	213.12b
	21	215.07±22.42d	236.51±21.38d	243.34±20.79d	231.64c
	Mean	209.64	214.54	211.46	211.88A£
MW-assisted hot air	9	179.44±17.30dδ	195.26±16.14d	260.41±42.79e	211.70a*
	15	184.61±21.68d	233.59±12.59e	313.17±26.15f	243.79b
	21	196.34±17.37d	240.19±23.67e	318.34±39.20f	251.62c
	Mean	186.80	223.01	297.31	235.70B

Mean±standard deviation. BSA, bovine serum albumin; MW, microwave.

* Means in the same column followed by the same lowercase letter are not significantly different (*p*<0.01) by Duncan's multiple range test.

δ Means in the same row followed by the same lowercase letter are not significantly different (*p*<0.01) by Duncan's multiple range test.

£ Means in the same column followed by the same uppercase letter are not significantly different (*p*<0.01) by Duncan's multiple range test.

## Conclusions

While percent DPPH scavenging activities ranged from between 63.24 and 95.10 in the MW roasting process, they varied between 62.98 and 94.78 in the MW-assisted hot air roasting process. The highest antioxidant capacity increased 160.4% at a 90°C roasting temperature and a 21-min roasting time using the MW roasting process compared to unroasted hazelnuts, while it increased 125% at 110°C and 15 min using the MW-assisted hot air roasting process. The maximum total phenolic content was obtained using the MW roasting process at 90°C for 21 min, whereas it was observed using the MW-assisted hot air roasting process at 110°C for 9 min. Hot air applied with an MW on hazelnuts in the MW-assisted hot air roasting process caused a lesser reduction in levels of resistant starch compared to the MW roasting process. Non-resistant starch particles were easily degraded into sugar during higher roasting times in both the MW and MW-assisted hot air roasting processes. Levels of protein concentration of hazelnuts roasted using the MW-assisted hot air roaster were higher than levels of hazelnuts roasted by the MW roaster under all the roasting conditions studied.
